# Entropy Signature for Crack Networks in Old Paintings: Saturation Prospectus

**DOI:** 10.3390/e20100772

**Published:** 2018-10-08

**Authors:** Juan César Flores

**Affiliations:** 1 Instituto de Alta Investigación IAI, Universidad de Tarapacá, Casilla 7-D, 1000000 Arica, Chile; cflores@uta.cl; Tel.: +(56 58)-2230-334; 2 Departamento de Física, Facultad de Ciencias, Universidad de Tarapacá, Casilla 7-D, 1000000 Arica, Chile

**Keywords:** cracks and old paintings, directional entropy, saturation, cracks samples

## Abstract

In desiccated films, particularly with old paintings, molecular bonds may break to create intricate patterns of macroscopic cracks. The resulting directions of the cracks quantifiably enable an evaluation of the entropy and degree of disorder in the network. Experimental tests on prepared samples and a two-interacting-variables model allow the evolution of entropy to be tracked. Calculations were performed, primarily using data from the painting *Girl with a Pearl Earring* by Vermeer, revealing that the left side of the girl’s face features a crack structure with higher entropy (or less order) than the right side. Other old paintings were considered. The extrapolation of experiments to these old paintings confirms that saturation still is not reached.

## 1. Introduction

Cracking within the paint layers of artwork are essential instrumental features in the recognition and authentication of masterpieces [1]. They are also part of a significant and unavoidable legacy. Crack topology is unique, and concerning forgery, challenging to reproduce. Additionally, these structures also develop in different types of systems [2,3]—for instance, in old potteries or even in the clays of the Atacama Desert (Chile) after robust El Niño (ENSO) phenomena. For some connections between entropy and strain energy, see References [4,5].

**Figure 1:**
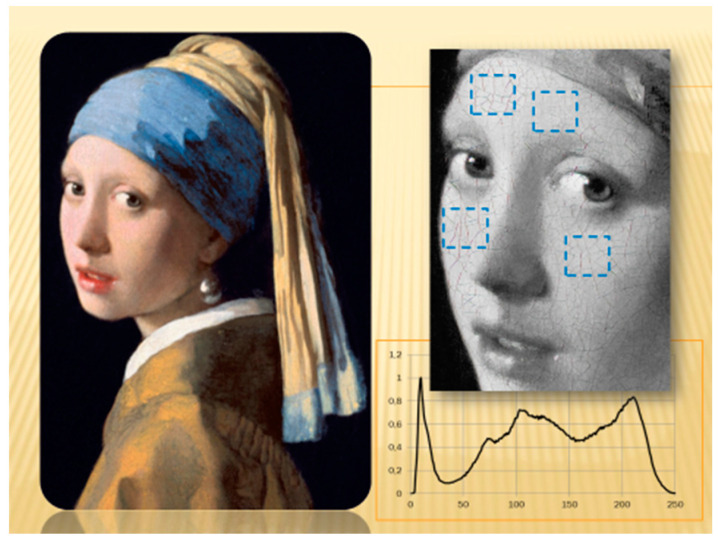
*Girl with a Pearl Earring* (J. Vermeer, Mauritshuis Museum). The blue squares approximately mark regions where crack histograms were constructed. The inset histogram corresponds to the relative color participation in the girl’s face (1 = black; 255 = white). Crack production still operates in this masterpiece (Section 6).

*Girl with a Pearl Earring* is an oil on canvas painted by Johannes Vermeer around 1665 A.D. It has inspired creative works including a novel (1999), a film (2003), and numerous expositions and cultural manifestations, including stamps. The Mauritshuis Museum website provides a digital image of the painting (Figure 1), which was downloaded and used as the main image in this study. The digital Image contains approximately 4000 × 4500 pxs^2^. Importantly, the painting includes intriguing disordered crack structures.

The girl’s face seems more illuminated on the right side than on the left, with the former also showing larger crack polygons. Using computational methods, Johnson et al. [6] performed an analysis of the direction of illumination for this painting. Pioneering experimental crack studies on pictures were also conducted by Mecklenburg [7] and Karpowicz [8], whereas Cornelis et al. [9] considered virtual restorations. Flores [10] constructed a mean-field elastic-mechanical-stress approach to study cracks in paintings and their correlations with film thickness. Experiments related to the relationship between crack areas and thicknesses are described in Ma and Burton [11]. In Reference [12], the crack evolution was studied in colloidal systems. References [13,14] considered the role of variable film thickness, and Romero et al. [15] studied the fracture control of films. As is well-known technically [16,17,18], bulk stress energy is the driver in cracking. Specifically, cracks develop when the bulk energy surpasses the fissure energy related to chemical bonds. Considerations from the theory of elasticity and cracks in master paintings also appear in [19,20].

This article is concerned with the inherent crack networks in old paintings, particularly with the directional entropy, the degree of disorder, and its production. From this point of view, every cracked masterpiece has a recognizable and quantifiable signature.

Section 2 is concerned with the definition of directional entropy. In fact, the operative idea is simple: for the histograms defining local crack directions in a given painting, the functional entropy will be explicitly evaluated in every case. Section 3 is briefly devoted to describing the image treatment by means of the free software package “ImageJ”. This package evaluates local directionalities by a gradient orientations method. Section 4 features a simple experiment that was used to obtain guidelines on crack dynamics and entropy production. A simple model of two interacting modes is fitted. In Section 5, histograms are presented and analyzed for Vermeer’s painting as well as other paintings. Explicitly, entropy is quantified in every case. Important asymmetries and the degree of the disorder are noted around the angles 0° and 90°. These asymmetries affect the entropy functional and become related to saturation loss. Section 6 is concerned with explicit comparisons between crack entropies in these paintings. The last section contains concluding remarks. Computational and experimental data support our conclusions.

## 2. Directional Entropy

In broad terms, a histogram of crack directionality is constructed by considering its frequency (amount) $f_{j}$ in a given angular inclination between $\theta_{j}$ and $\theta_{j}+\Delta \theta$, where $0\leq f_{j}\leq 1$. Considering $\Delta \theta =1^{{}^{\circ}}$ and, in analogy to the thermodynamic concepts of Gibb’s entropy [21,22,23,24] and information [21,25,26,27], the associated crack directional entropy is

(1) $$S_{180}=-\frac{1}{Log\left(180\right)}\left(\overset{j=+135}{\underset{j=-45}{\sum }}f_{j}Log\left(f_{j}\right)\right).$$

The range selection for “angles” $-45^{{}^{\circ}}\leq j\leq +135^{{}^{\circ}}$ was explicit in order to show the behavior of the specific frequencies $f_{90^{{}^{\circ}}}$ and $f_{0^{{}^{\circ}}}$ in the painting histograms. In the following, the sub-index denoting the entropy will be drop (i.e., $S=S_{180}$). Although the log-function is with base 10, the function *S* is independent of the base. As an example, for a perfect two-dimensional square network with $f_{90^{{}^{\circ}}}=0.5$ and $f_{0^{{}^{\circ}}}=0.5$, the associated entropy is S≈0.134. In contrast, as with the microcanonical ensemble [23,24,25], when all frequencies $f_{j}$ are equal, the entropy is at its maximum value S=1. Thus, a flatter histogram has more entropy whereas one with pronounced peaks has lower entropy.

Two notes related to Equation (1):

It measures the structural disorder of cracks in a given sample, as entropy and disorder are considered equivalent.

It is related to frequencies $f_{j}$ and not probabilities $p_{j}$. In information theory, the usual functional requirements of entropy suggest considering the practical frequency rate $p_{j}=n_{j}/\sum n_{j}$ (with $n_{j}$ integer) [25].

## 3. Image Treatment

For this work, the software package “ImageJ” (attainable online, https://imagej.net/ImageJ) was the tool of choice to analyze cracks in paintings. The item *Directionality* is of particular use for producing histograms. For every studied region of the painting, the followed operational procedure is (1) Analysis. (2) Directionality. (3) Local gradient orientation. (4) Nbins: 180. (5) Histogram start: −45.

Directionality calculations, technical concepts, and usage can be viewed on the web page of ImageJ. Mainly, it uses the local gradient orientation method to build the histograms. Histograms exhibit the collection of directions for different previously specified angles (number of bins, ranges, and others). The technical aspects of this method can be revised in References [28,29]. 

Finally, the sizes of the chosen pixeled squared regions used in every painting were variable and depended on the quality of the obtained image. The sizes ranged between 250 × 250 to 500 × 500 pxs^2^.

## 4. Experiments on Entropy Growth and Saturation Condition

### 4.1. Experiments on Tapes

The experiments described herein allow the entropy dynamics in paintings to be described and compared. Further, a simple model can explain the evolution of the central peaks of painting histograms. Cracks are universal phenomena [11], and many ways exist to describe and analyze them. Here a brief description of the utilized materials is now presented. Materials are easily obtained in the market. The primary material used was ordinary commercial nail varnish, mostly composed of nitrocellulose, toluene, and formaldehyde. Kaolin and ethanol served as additional primary products to create the cracks, as these products rapidly evaporate. The substrate was a transparent adhesive tape that held the varnish film firmly. This substrate resembles canvas more than it does a hard material like wood. A drying varnish film of approximately 1 × 1 cm^2^ was used. In this way, a sequence of 35 photographs were taken at 20-s intervals as the film dried. This system appeared to saturate around 700 s, with no new visible cracks appearing.

**Figure 2:**
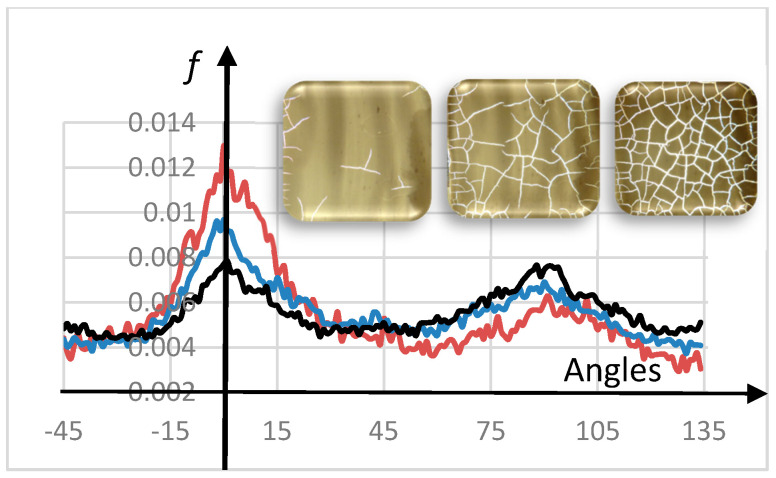
Three histograms showing experimentally how crack directionality (amount *f*) changes in time. The red curve is related to the early stages of cracks. Blue is 100 s after red, and black (saturation) 500 s later. The insets show three photographs (out of a total of 35) near the beginning, the middle, and saturation points. The full sequence of images enables the evolution of crack entropy to be tracked. Apart from the fluctuations, in saturation, the two central peaks equalize over time (black curve).

**Figure 3:**
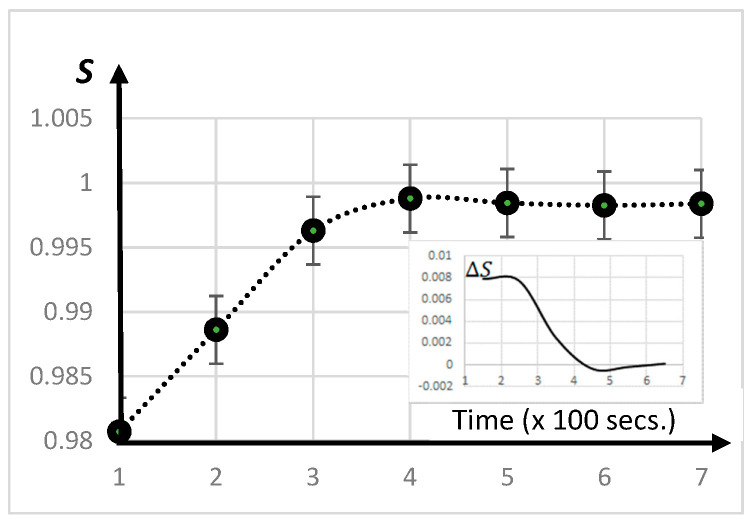
Evolution in crack entropy obtained from a sequence of images of drying varnish. A saturation in crack entropy is at a value of around 0.998 when both peaks in Figure 2 become equivalent (apart from noise fluctuations). The data points were taken 100 s apart. This experiment also typifies entropy production in cracked paintings. The inset shows the entropy variation ΔS as a function of the time corresponding to a damped dynamics.

Figure 2 shows three histograms of the amount of crack directionality *f*. In the same figure, three representative photographs at different stages of desiccation are also presented. The red histogram is related to the early stages at which only a few cracks are found. The blue histogram is associated with an intermediate step at 100 s later. The black, taken 500 s from the blue curve, is typical of the saturation stage. Figure 3 shows the evolution of crack entropy for a sequence of 7 out of the 35 photographs (separated by 100 s).

Importantly, from Figure 3, the entropy evolves, showing a definite growth in the early stages and then reaching a saturation point when ignoring fluctuations, both natural peaks at 0° and 90° tend to be equilibrated (black curve, Figure 2).

Based on the experiments, three comments are relevant:

A diffusive behavior for the frequency *f* around 0° and 90° seems to operate. The dynamics corresponds to the fall of one peak and the growth of another (Figure 2).

With time, discounting fluctuations, a saturation regime for crack entropy holds when the two central peaks become equivalent (Figure 2). Additionally, the dynamics becomes like a damped system (Figure 3). 

The experiments on drying varnish suggest that the entropy production occurs similarly as typically encountered in thermodynamic systems.

### 4.2. A Two-Interacting-Peak Model

From the temporal evolution for the entropy in Figure 3, the difference $\Delta S=S_{j+1}-S_{j-1}$ is computed. The inset in Figure 3 shows the experimental variation of ΔS as a function of time. There were damped oscillations before reaching the saturation point at approximately *S* = 0.998. These oscillations were related to the interactive dynamic between the two peaks in Figure 2. Consequently, a two-mode model can be used—that is, like the dynamics of two equal masses interacting by a spring.

Consider the entropy function defined as:

(2) $$S_{2}=-\frac{1}{Log2}fLog\left(f\right)-\frac{1}{Log2}gLog\;\left(g\right),$$

where

(3) f+g=1,

and the entropy variation becomes

(4) $$\frac{dS}{dt}=\frac{1}{Log\left(2\right)}\frac{Log\left(g\right)}{Log\left(f\right)}\frac{df}{dt}$$

The experimental saturation point where S≈0.998 corresponds to f≈0.476. Thus, Equation (4) around this point becomes

(5) $$\frac{dS}{dt}=2.892\frac{df}{dt}.$$

The damped oscillations of ΔS in the inset graph of Figure 3 can be well modeled by ΔS~0.15e−t0.9sin(2πt5−2.2), where 2<t<7, corresponding to a period T ~ 5×100 (s) and a decay rate of γ ~ 1.1×100 (1/s).

## 5. Crack Networks in *Girl with A Pearl Earring* and Other Paintings

In this section, histograms for Vermeer’s painting (Section 5.1), Petrus Christus’ *Portrait of a Young Woman*, and Rogier van der Weyden’s *Portrait of a Lady* (Section 5.2) are presented. Comparisons and similitudes of crack patterns in these masterpieces are explicitly displayed. Generally, pairs of sites (left and right face, with respect to the nose, Figure 1) are shown to emphasize the effect of face lightness and the consequences of network crack formation. Directional entropies are calculated using Equation (1) and are compared in each case (Table 1 and Section 4).

### 5.1. Entropy Cracks in the Girl’s Front and Cheeks

**Figure 4:**
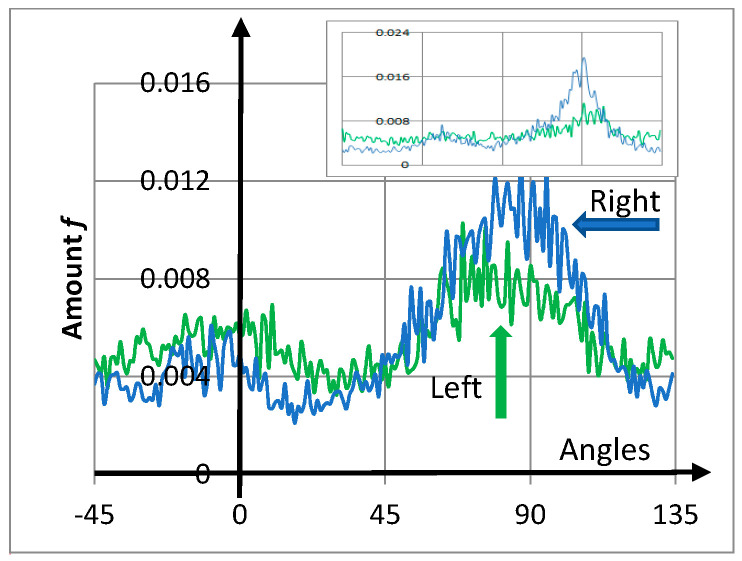
Vermeer’s painting. Histograms of the amount of directional cracks *f* for the girl’s right (blue) and left (green) front side as a function of crack inclination angles (from −45° to 135°). The entropy of the right front side was less than that on the left, which is graphically flatter. The inset in the top-right corner shows the amount of directional cracks in the girl’s cheeks as a function of angle. Additionally, for all curves, the central peaks at 0° and 90° were not symmetric, indicating that saturation was not reached (Section 4 and Section 6).

Obtained from the right and left front sides of the girl’s face in Vermeer’s painting, Figure 4 shows the corresponding histograms for the number of cracks *f* as a function of angle ranging from −45° to 135°. A square of 250 × 250 pxs^2^ was selected for every region (Figure 1). Both histograms presented broad maximum peaks at approximately 0° and 90°, showing privileged directions related to the rectangular boundary conditions for the stress tensor. Nevertheless, crack-angles pointed in all directions. Note that the two peaks were not symmetrically arranged as in the experiments of Section 2. Additionally, the entropy values were comparable (particularly the right-blue curve) with the experiments.

From data related to the histograms, the entropy was evaluated numerically, obtaining $S_{R-front}=0.979$ for the right side and $S_{L-front}=0.994$ for the left. Consequently, the girl’s left-hand front side (green) contained quantitatively more disordered cracks than the right-hand front side (blue). This quantifiable difference was related to the comparatively large blue peak at around 90°. Note the high degree of disorder when compared with the perfect square network S≈0.134.

The inset in Figure 4 presents the corresponding histograms for the girl’s right and left cheeks (250 × 250 pxs^2^). Values of entropy are given in Table 1.

From calculations, cracks in the right-hand side of the girl’s face were more regular than those on the left. The origin of this asymmetry is related in part to the illumination effect of the girl’s face on the right-hand side, requiring a greater thickness of paint-material. 

### 5.2. Portrait of A Young Woman by Petrus Christus

**Figure 5:**
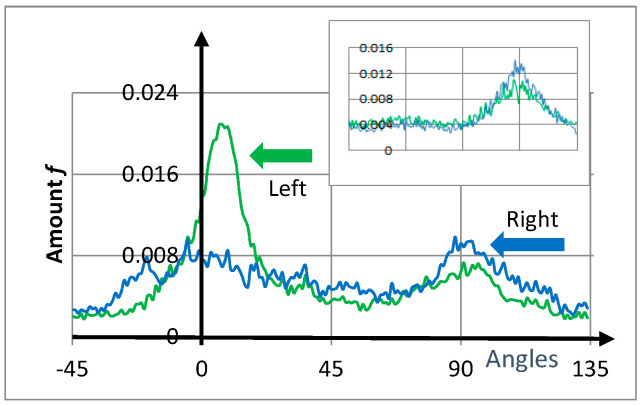
The amount of directional cracks *f* for the *Portrait of a Young Woman*. The entropy obtained for the right cheek was larger than that for the left because of the pronounced (green) peak. Moreover, when viewed against Vermeer’s painting, the cracks in the left cheek appeared more ordered than those in the right cheek. Inset: histograms of *Portrait of a Lady* by Rogier van der Weyden. As for the previous examples, the central two peaks of the curves were not symmetrical.

This masterpiece on wood dates from around 1465 A.D., and contains a series of remarkable and evident cracks. In particular, in the image (500 × 500 pxs^2^), the crack-cells in the right cheek were larger than those in the left cheek, yet the latter was more ordered than the previous (pronounced peak around 0°, Figure 5, green). The crack entropy values are given in Table 1.

### 5.3. Portrait of A Lady by Rogier Van Der Weyden

This painting on wood dates approximately from 1460 A.D. In this case, the histograms for the crack directions seemed almost similar for the right and left cheeks (Figure 5, inset, 250 × 250 pxs^2^). Nevertheless, the configuration of the cracks was slightly more ordered on the right-hand side than on the left (Table 1).

## 6. Comparing Results for Old Paintings and Experiments

Table 1 compares the entropies obtained from the left and right sides of the faces in the pictures under study. The right-hand side of the face in Vermeer’s painting displayed a more ordered network than the left side, whereas the opposite applied to Petrus Christus’ portrait. Table 1 also lists the estimations of the relative changes in left and right entropy ΔS/S.

Additionally, for Vermeer’s painting, we evaluated the entropy of two regions from the blue turban (250 × 250 pxs^2^, last row of Table 1). As in Christus’ painting, the entropy on the left was smaller than on the right. Nevertheless, the region around the turban was quite disordered with blurry lines between cracks and painting. Indeed, the turban was painted with a particular oil technique. Consequently, this result warrants caution.

Importantly, in this work, the central peaks were not symmetrical in the eight histograms (Figure 4 and Figure 5). Specifically, ignoring the noise fluctuations, no reflection symmetry was present around the 45° axis. These results on the eight graphs, compared with the experiments described in Section 2 (Figure 2), tell us that saturation is not still reached in these masterpieces.

## 7. Conclusions

Crack structures not only reveal authenticity, but also disclose the history of the painting. This work promotes the idea that crack entropy is a useful tool in the characterization of old paintings’ dynamics.

Experiments suggest that crack entropy grows in time in desiccated films (entropy production). The two initial asymmetric privileged peaks around 0° and 90°, related to boundary conditions, remain but tend to become equivalent (Figure 2). Because the two central peaks (Figure 4 and Figure 5) are asymmetric in the considered paintings, the main conclusion is that a long crack progression path remains for these masterpieces.

In forthcoming work, other complementary applications of entropy as a tool (crack cell areas, crack cell perimeters, color distribution histograms, among others) will be considered. Indeed, the fractal dimension [30,31] of crack topology is not discarded. When applied, this set of tools seemingly defines the signature of a given painting or perhaps even the individual signature of the artist. Crack propagation velocities and energy absorption [32] will be considered in further studies in addition to particular human-made hexagonal topologies [33].

## Figures and Tables

**Table 1:** Entropy comparisons between the masterpieces studied. The last column describes the relative change in entropy. The last row corresponds to the entropy of cracks with blurry lines in the girl’s turban. From the data, note that a more ordered crack structure (less entropy) was encountered in the left cheek of the woman in Christus’ painting (Young woman). In contrast, the girl’s turban (Vermeer) had the more intricate pattern.

	Right Entropy	Comparison	Left Entropy	|ΔS/S|
Girl’s front (canvas)	≈0.979	<	≈0.994	0.015
Girl’s cheeks (canvas)	≈0.966	<	≈0.995	0.029
Young Woman (wood)	≈0.990	>	≈0.957	0.033
Portrait of a Lady (wood)	≈0.978	<	≈0.990	0.012
Girl’s turban (canvas)	≈0.998	>	≈0.997	0.001
